# Research on gradual changes in incomplete technical information and optimization mechanism of innovation factor allocation efficiency

**DOI:** 10.1016/j.heliyon.2024.e31440

**Published:** 2024-05-17

**Authors:** Linsheng Yang, Bing Jiang, Qi Xu

**Affiliations:** a New Economics Institute, Ningbo Polytechnic, No. 388, Lushan East Road, Beilun District, Ningbo, 315800, Zhejiang, China; b Taizhou Economic Research Institute, Taizhou Vocational and Technical College, Taizhou, 315800, Zhejiang, China

**Keywords:** Innovation factors allocation, Input-output efficiency, Technical information, Optimization mechanism, Software development listed companies, Processing and manufacturing listed companies, GMM regression analysis

## Abstract

The innovation factor allocation efficiency (IFAE), indicated by R&D institutions' technical input information and manufacturers’ technical output information, is mainly optimized by transformation of incomplete technical information into relatively complete information and finally complete technical information. There are two optimization mechanisms, namely single optimization and continuous optimization. The empirical analysis selected data from 73 listed companies in software development and 596 listed companies in processing and manufacturing, and testified its hypotheses with GMM regression results. The research finds that the differences in benefits are caused by gradual changes in technical information. With shared goals of benefit maximization between R&D institutions and manufacturers, a correct strategy for technical information can optimize the IFAE. Single optimization refers to the one-time recognition or identification of relatively complete technical information and complete information, acquisition of technical information from one-time cognition, and thorough application of resource input, thus realizing high-level technology progress gradually. Continuous optimization, based on single optimization, involves the prioritized reporting of complete technical information by participants, thus achieving optimal IFAE. Therefore, it is necessary to accelerate the improvement in the incentive mechanisms for technology innovation efficiency. R&D institutions should promote original innovation and integrated innovation, expand technical information increment, deepen the cooperation with manufacturers actively, and facilitate the technical information sharing.

## 1 Introduction

Considering that China's economy is turning to a high-quality development stage, there is a need to improve its development quality, enhance its development efficiency and reform its driving forces [[1], [2], [3]]. Improving the efficiency of innovation factor allocation and fully releasing the potential of innovation factor allocation are important driving forces for realizing high-quality economic development [4]. Optimization of innovation factor allocation efficiency (IFAE) is the main direction of efficiency enhancement and depends on the following aspects: the possibility of reporting technically input information by R&D institutions and technically output information by manufacturers, and the differences with their actual technical information respectively [5]. From the input process to the output process, when incomplete technical information is gradually transformed into relatively complete and then complete technical information, the gaps between reported technical information and actual technical information narrow down gradually until they finally disappear, and hence the IFAE is optimized [6]. However, there are many issues to be studied at the theoretical level, such as the specific characteristics of technical information changes during the process of innovation factors allocation, the manners of transmission and transformation of technical information from the input process to the output process, the methods used by R&D institutions and manufacturers to determine the probability of realizing each other's benefit maximization goal, the differences between single and continuous optimizations of IFAE, the ways of functioning of such optimization mechanisms, and selection of proper technical information-related strategies adopted by R&D institutions and manufacturers to achieve the optimal IFAE.

This study focuses on the analysis and comparison of single optimization and continuous optimization of IFAE in terms of their preconditions, goals, probability judgment and selection of information strategies. The single optimization mechanism of IFAE means that R&D institutions and manufacturers try to recognize and identify their own actual technical information at a certain probability, and, incentivized by their goals of benefit maximization, report more complete technical information once to reduce the discrepancies with the actual information, and then determine the probability of the other party's reporting complete technical information to choose optimal technical information-related strategies, so as to improve the IFAE. In contrast, the continuous optimization mechanism of IFAE consists of multiple optimizations where R&D institutions and manufacturers, based on their actual technical information, report technical information many times. Each single optimization brings about more complete information than the previous one, gradually reducing the discrepancies with the actual technical information. Consequently, each side obtains a more accurate understanding of the other side's technical information, and acquires relatively more complete and finally complete technical information, thus optimizing the IFAE.

The originality of this study is that it compares and analyzes the different paths of single optimization and continuous optimization of the IFAE. The results find that the technical input information of R&D institutions and the technical output information of manufacturers can be collected only in testing and production processes, without a spillover/loss or diffusion/amplification. The generation and reporting of technical information are completed successively, rather than simultaneously. There are many options for information strategies. After continuous and multiple identifications, transmissions and judgments of technical information, an efficiency optimization mechanism is established to obtain complete technical information gradually, thus optimizing the IFAE.

In terms of theoretical contributions, this paper analyzes the technical information-related strategies selected by R&D institutions and manufacturers under the guidance of their benefit maximization goals, and compares the differentiated methods of single optimization and continuous optimization for recognizing and absorbing technical information and determining the probability. Multiple recognitions of incomplete technical information can help obtain relatively complete and finally complete technical information, thereby causing the changes in IFAE. With respect to the practical contributions, this study explores a better optimization path of IFAE. Specifically, multiple single optimizations could help acquire more complete technical input information and technical output information, avoid the diffusion and spillover of incomplete technical information, reduce the idleness and waste of innovation factors, improve the intensive utilization of innovation factors, and further provide reference for the government to improve technology legislation, increase intellectual property protection, encourage collaborative innovation between industry, academia, and research, and fully leverage the role of enterprises as the main body of innovation.

## 2 Literature review

### 2.1 Characteristics of technical information changes during the allocation of innovation factors

Technical information changes are closely related to the allocation of innovation factors. China's industrial enterprises are not yet at an advanced level in terms of technology. The information asymmetry leads to a reduction in IFAE [7]. Under the condition of complete technical information, the market can allocate innovation resources at an optimal efficiency [8]. Financial market information is included, as a variable, in the standard macroeconomic model to reflect the changes in capital factors allocation and their influence on the total output level [9]. Firms that operate in high-velocity markets must configure and utilize diverse technological innovation capabilities. S. Sinan Erzurumlu and Nathan Smith [10] examine the configuration of a high tech firm's technological innovation capabilities involving R&D, manufacturing and marketing capabilities to develop technology exploring or market exploiting activities in response to shifts in the customer value trajectory. More efforts could be made to analyze technical information in detail, explore the changes in technical information at different levels of completeness, and study the changes in benefits of IFAE and the efficiency optimization mechanisms for innovation factors allocation.

### 2.2 The relation between technical information at different levels of completeness and the benefits of innovation factors allocation

The levels of completeness of technical information, namely incomplete, relatively complete and complete technical information, have an impact on the benefits of innovation factor allocation. Incomplete technical information, affected by the ownership, industries or regions, leads to the misallocation of innovation factors, resulting in a large loss of TFP for listed industrial companies [11]. The stock price reflects the degree of incompleteness of technical information and helps to identify the changes in factor allocation structure [12]. More real-time information is required to calculate the expected benefits under incomplete technical information through the probability distribution function [13]. Based on the above-mentioned literature, this study will focus on the selection of technical information strategies by R&D institutions and manufacturers because different strategies bring different IFAE and input-output ratios.

### 2.3 Selection of technical information strategies by R&D institutions and manufacturers

The combination of technical information strategies of R&D institutions and manufacturers constitutes the optimization mechanism of IFAE. Utilizing TFP dispersion and covariance to describe the degree of resource mismatch, Chinese industrial enterprises are found to have resource mismatches of varying degrees [14]. Mol [15]believes that the input of innovation factors will react to the technical information of manufacturers. Complete technical information can optimize the input-output efficiency, and properly selected strategies enable innovation factors to more fully integrate with physical-form factors. Based on the data about German industrial manufacturing, the study considers that manufacturers can reduce the expected deviation in the future by choosing complete technical information strategies [16]. On the basis of existing literature, this study will analyze the selection of technical information strategies by R&D institutions and manufacturers, and how different technical information strategies motivate participants to give priority to reporting complete technical information when the benefit objectives of the two sides are consistent.

### 2.4 Optimization mechanisms of IFAE

The optimization mechanism of corporate technical efficiency includes such internal factors as the technical R&D level, governance structure and factors allocation [17]. There is a structural change between the overall output level of processing manufacturers and technical information, and micro technical innovation activities can obtain more complete technical information [18]. Complete technical information can significantly improve the factor allocation ratio, enabling the input-output efficiency to approach the optimal level [19]. Boyan, Jovanovic [20] thinks that incomplete technical information hinders the improvement of IFAE, negatively affects the optimal combination of factors, resulting in idleness, waste or shortage of factors, and makes the technical factors (in the forms of knowledge and information) unable to match material factors and human capital investment effectively. Technical information can be used to measure the allocation of innovation factors and reflect the changes from the input process to the output process of innovation factors. The conditions for realizing the optimization mechanism of IFAE include the established elasticity of factor substitution, constant returns to scale, inclusion of all the surplus in the production efficiency, and absence of capital adjustment costs [21]. Some Chinese scholars used the data about Chinese manufacturing enterprises from 1998 to 2013 to estimate the efficiency of resource allocation. They found that there is a huge potential for improving the resource allocation efficiency in China and enterprise heterogeneity is an important influencing factor for the efficiency [22]. Scholars found that improving the efficiency of innovation factor allocation is one of the key to solving many problems in China's sustained high-quality development process [23]. The above literature studied the changes in technical information and benefits during the allocation of innovation factors, the selection of technical information strategies by both sides, and optimization mechanisms. This paper will further study the optimization mechanism of IFAE, incorporate incomplete technical information into the analysis of optimization mechanisms, and analyze how to obtain complete technical information more accurately, so as to optimize the input-output structure and improve the IFAE.

The subsequent parts of this paper are stated as follows: part 3 refers to theoretical model analysis; part 4 deals with industries, indicators and data processing; part 5 involves analysis of empirical results; and the final part gives conclusions and policy recommendations.

## 3 Theoretical model analysis

### 3.1 Characteristics of changes in technical information during the innovation factor allocation

#### 3.1.1 Detailed analysis of technical information and innovation factors allocation

This paper expands the mechanism design model [24] and assumes that the innovation factors allocation involves two participants. One participant 0 refers to an R&D institution, and its technical input information includes two items: innovation factor input F and marginal cost mc. When the actual technical input information of an R&D institution is (F=1,mc=1), complete technical input information will be generated if the reported information is (F=1,mc=1), while incomplete technical input information will be generated if the reported information is (F=0,mc=0).Similarly, when the actual technical input information of an R&D institution is (F=0,mc=0), complete technical input information will be generated if the reported information is (F=0,mc=0), while incomplete technical input information will be generated if the reported information is (F=1,mc=1). The other participant i refers to a number of manufacturers (the number ranges from 1 to n). i∈N≡{1,⋯,n} represents manufacturers in the allocation of innovation factors, and o represents their technical output information. The generation process of complete and incomplete technical output information is similar to that of R&D institutions. s refers to the supply-demand relationship of technical achievements in the allocation of innovation factors, $s_{i}^{p}$ represents that the R&D institution transfers the value of technical achievements to the manufacturer, and $s_{i}^{a}$ means that the manufacturer pays for technical achievements to the R&D institution. The output results of R&D institution and manufacturers are collected as $m=\left(m_{0},\cdots ,m_{n}\right)\in M\equiv {\left\{0,1\right\}}^{n+1}$.

There are two preconditions for realizing the allocation of innovation factors: a) an R&D institution receives a payment higher than its R&D cost, namely $\sum_{i\in N}s_{i}^{a}\geq m_{0}F$; b) the manufacturer i acquires the value of technical achievements greater than its payment for the technical achievements, namely $s_{i}^{p}$ >$mc\sum_{i\in N}m_{i}$. The two preconditions can be expressed as follows.

(1) $$\sum_{i\in N}s_{i}^{a}+s_{i}^{p}\geq m_{0}F+mc\sum_{i\in N}m_{i}$$

According to equation (1), it can be seen that the R&D institution reports complete technical input information, and the manufacturer reports complete technical output information. The benefits of each party exceed its costs, indicating that the allocation of innovation factors produces value-added effects. As the representation of knowledge-form achievements, technical information can be put into use repeatedly without spillover or loss, and be transferred to the input-output process, so as to improve the input-output ratio of innovation factors continuously.

The IFAE is the ratio between the investment costs of R&D institutions' technical innovation activities and the output value of manufacturers' new products [25]. From the perspective of changes in and identification of technical information, (F,mc,o) is composed of R&D institutions' technical input information (F,mc) and manufacturers’ technical output information o, by matching the input process and output process during the allocation of innovation factors. R&D institutions invest some innovation factors and then obtain corresponding technical achievements which will be later allocated to manufacturers' processing and manufacturing processes. The process of producing related new products constitutes the optimal allocation of innovation factors a∈A.The allocation of innovation factors is defined as $a^{*}\left(F,mc,o\right)=\left(s^{*}\left(F,mc,o\right),m^{*}\left(F,mc,o\right)\right)$.

#### 3.1.2 The relation between the changes in technical information of R&D institutions and those of manufacturers

In the allocation of innovation factors, if the R&D institution reports complete technical input information, the manufacturer will be considered to have reported complete technical output information, which is expresses as follows:

(2) $$m_{0}\left(F,mc,o\right)=1\Rightarrow \exists i\in N:m_{i}\left(F,mc,o\right)=1$$

According to logical reasoning of equation (2), when $m_{i}\left(F,mc,o\right)=0$, $m_{0}\left(F,mc,o\right)=0$. As the IFAE reaches its optimal level and the manufacturer i's output information contains incomplete technical information, the R&D institution's technical achievements include incomplete technical input information. The reasoning results show that complete technical input information and complete technical output information are the preconditions for the optimization of the IFAE. The technical input information is transferred to the processing and manufacturing processes through the allocation of innovation factors to realize complete transformation, without any amplification or loss caused by a spillover. The IFAE does not deviate from the optimization state because of the changes in technical information. If incomplete technical input information or incomplete technical output information exists, the manufacturer fails to fully absorb the technical achievements of the R&D institution and the low input-output ratio means a waste of the invested innovation factors, resulting in a deviation of the IFAE from the optimal state [26]. Hence, hypothesis 1 is stated as follows:

Hypothesis 1 When R&D institutions and manufacturers absorb and transform technical output information and technical input information respectively, there is no spillover or loss of their complete information or amplification of incomplete information. They can gradually obtain complete technical information only through experiments, perception, recognition, etc.

### 3.2 Surplus of innovation factor allocation and changes in complete technical information

A single allocation (a) of innovation factors is included in a set (A) of continuous multiple allocations, that is a∈A. The value added from technical output information $o_{i}$ of the manufacturer i is $R_{i}\left(a|o_{i}\right)=o_{i}m_{i}-s_{i}^{a}-s_{i}^{p}$. The value added in the output process is the benefit $o_{i}m_{i}$ from the manufacturer's application of the transformed technical information, net of the payment ($s_{i}^{a}$) for technical achievements and the value ($s_{i}^{p}$) of technical achievements transferred by the R&D institution. The added value realized by the R&D institution through technical input information (F,mc) is $\Omega \left(a|F,mc\right)=\sum_{i\in N}\left(s_{i}^{a}+s_{i}^{p}-m_{i}mc\right)-m_{0}F$. The benefit of the R&D institution comes from the value of technical achievements transferred to the manufacturer, net of related R&D costs and the payment by manufacturers for technical achievements. The allocation of innovation factors is jointly facilitated by one R&D institution and a number of manufacturers. With the established IFAE (F,mc,o), the surplus of technical achievements m is shown as follows:

(3) $$D\left(m|F,mc,o\right)=\sum_{i\in N}\left[o_{i}-mc\right]m_{i}-m_{0}F=\Omega \left(a|F,mc\right)+\sum_{i\in N}R_{i}\left(a|o_{i}\right)$$

According to equation (3), the surplus of innovation factor allocation is the sum of the added value from complete technical input information of R&D institutions and the added value from complete technical output information of numerous manufacturers. The optimal allocation efficiency of innovation factors means a balance between the input resources of R&D institutions and the output value of manufacturers, and the surplus level tends to be zero. When manufacturers report the complete technical output information, it will be fully transformed and transferred to the R&D institution, indicating the full transformation of complete technical output information. There is no waste or gap of technical information in the allocation of innovation factors. Hence, hypothesis 2 is stated as follows.

Hypothesis 2 When incomplete technical information is gradually transformed into relatively complete and finally complete technical information, the IFAE is optimized, and the benefit growth mainly depends on the continuous acquisition of technical information. The full absorption and transformation of complete technical information can significantly increase its contribution to the benefits and maximize the surplus of innovation factor allocation.

### 3.3 Allocation of innovation factors and selection of strategies for technical information

During the allocation of innovation factors, R&D institutions have two information strategies, i.e. incomplete and complete technical input information, and manufacturers also have two strategies, namely incomplete and complete technical output information. The two parties choose different proper technical information strategies for pursuing benefit maximization. A set of strategy selection rules constitutes the optimization mechanism of IFAE, whose optimal state is the Bayesian perfect Nash equilibrium of the game between the two sides. The complete technical input information $\left(F^{r},mc^{r}\right)$ and the complete technical output information $o^{r}$ reported by both sides respectively are from the technical input information set *I* and the technical output information set *O* respectively, which are consistent with the actual technical information, so that the IFAE is optimal. The selection of technical information strategies by both parties depend on the correlation between the coverage of complete technical input information of R&D institutions and the number of manufacturers when their benefit maximization goals are realized.

In terms of continuous optimizations of IFAE, after the processes such as cognition, identification, absorption and transformation, complete technical information is gradually realized where each single optimization λ=(s,m) is effective.

(4) $$\left(s_{i}^{a}\left(F,mc,o\right),s_{i}^{p}\left(F,mc,o\right)\right)=\left\{ \begin{array}{c} \left(o_{i}F/n\left(o\right),o_{i}\left[1-F/n\left(o\right)\right]\right)ifT\left(F,mc\right)<n\left(o\right)\\ \left(0,0\right)ifT\left(F,mc\right)>n\left(o\right) \end{array}\right.$$

According to equation (4), in light of the R&D institution's technical information strategy, when the coverage of technical input information T(F,mc) is lower than the number of manufacturers n(o) at the time of realizing the goal of maximizing benefits, there is a greater market demand for technical information. Specifically, more manufacturers need to absorb and apply technical input information, and fully transform complete technical input information, so the technical output information reflects the technical status of actual production results in a more comprehensive way, giving priority to reporting technical input information. The payment by manufacturers for technical achievements to the R&D institution is represented by $s_{i}^{a}\left(F,mc,o\right)$, the actual value of technical achievements transferred by the R&D institution to manufacturers is expressed by $s_{i}^{p}\left(F,mc,o\right)$, and the output values are indicated by $\left[o_{i}F/n\left(0\right),o_{i}\left(1-F/n\left(0\right)\right)\right]$ respectively. When the coverage of technical input information T(F,mc) is greater than the number of manufacturers n(o) at the moment of achieving the goal of benefit maximization, it is difficult for technical achievements to be fully integrated into the allocation of innovation factors, leading to a low demand for technical achievements, technical information's failure to be fully identified, and impossibility of complete absorption and transformation of technical achievements and acquisition of expected returns from complete technical information. Thus, the manufacturers' payment for the technical achievements $s_{i}^{a}\left(F,mc,o\right)$ and the value created by the transformation of technical achievements of the R&D institution $s_{i}^{p}\left(F,mc,o\right)$ are 0. Hence, hypothesis 3 is proposed as follows.

Hypothesis 3 Under the influence of benefit maximization, when the coverage of technical input information of the R&D institution is lower than the number of manufacturers, there is great potential for application and transformation of technical input information. Both parties report complete technical information, absorb and transform technical information, and make full use of the complete technical information, thus resulting in the optimal IFAE level. Otherwise, the share of technical information and its absorption and transformation will be zero, and the allocation of innovation factors will stop.

### 3.4 Optimization mechanism of IFAE for pursuing benefit maximization

#### 3.4.1 R&D institutions and manufacturers shall give priority to reporting complete technical information

In terms of the single optimization mechanism of IFAE λ, the precondition for the R&D institution to report complete technical input information $m_{0}\left(F,mc,o\right)=1$ is the prioritized full payment by manufacturers for technical achievements and the R&D institution's reporting of the technical input information based on the payment by manufacturers for the achievements to avoid value spillover due to excessive reporting of information. The benefit maximization goal of R&D institutions is shown as follows:

(5) $$\Omega^{\lambda }\left(F,mc\right)=\left\{T|\sum_{i\in N}s_{i}^{a}\left(F,mc,o\right)=T*m_{0}\left(F,mc,o\right)=1\right\}$$

According to equation (5), when manufacturers give priority to paying full price $\sum_{i\in N}s_{i}^{a}\left(F,mc,o\right)=T$ for technical achievements, this will help the R&D institution achieve its goal of benefit maximization and tend to report complete technical information. If the manufacturers fail to fully pay for the achievements, the R&D institution will report part of or none of the technical information, according to the principle of “reporting information only when obtaining equivalent benefits”, and will not report technical information that will create the value exceeding its own benefit. Importantly, the R&D institution needs to accurately determine the probability of achieving the goal of benefit maximization after the manufacturers obtain technical information. Manufacturers gain the added value from the transformation and application of technical achievements, and its goal of benefit maximization is expressed as follows:

(6) $$R^{\lambda }\left(O|F,mc\right)=\left\{T|m_{0}\left(F,mc,o\right)=1*\sum_{i\in N}s_{i}^{a}\left(F,mc,o\right)=T\right\}$$

According to equation (6), the manufacturers pay for technical achievements to achieve its goal of benefit maximization, on the premise that the R&D institution gives priority to reporting complete technical input information $m_{0}\left(F,mc,o\right)=1$. The manufacturers pay the price equal to the value of reported information according to the completeness level of the technical information reported by the R&D institution, and gradually realize the goal of benefit maximization. If the value of technical input information is underestimated, the payment for technical achievements will decline. If the value is overestimated, the manufacturers will not accept the high price, leading to a suspension of the allocation of innovation factors. This will depend on whether the manufacturers can accurately identify, absorb and apply the technical input information of the R&D institution and then determine the probability of realizing benefit targets by the latter. Thus, hypothesis 4 is raised as follows.

Hypothesis 4 With the gradual change of incomplete technical information into complete technical information, the IFAE tends to be optimized, and its continuous optimization mechanism is realized on a basis of countless single optimization mechanisms. In the case of incomplete technical input information and complete technical output information, the R&D institution reports the complete technical input information without increasing R&D costs, thus contributing to the full utilization of innovation factors and significant improvement in the IFAE. In the case of complete technical input information and incomplete technical output information, manufacturers report complete technical output information, deeply tap the potential capacity of established investment of innovation factors, and optimize the IFAE. In the case of complete technical input information and complete technical output information, each improvement in the allocation of innovation factors can neither reduce the investment costs of the R&D institution nor enhance the output level of any manufacturer, and the IFAE reaches the optimal level.

#### 3.4.2 The R&D institution and the manufacturers determine the probability of the other party's achieving the goal of benefit maximization

When a large number of single optimization mechanisms constitute a continuous optimization mechanism, according to the principle of probability multiplication, the probability of the manufacturers' achieving the goal of benefit maximization is $\sum_{j\in J}\sum_{o\in O^{r}}k_{j}\varepsilon \left(o\right)$, where $k_{j}$ refers to the shares from the transformation of technical output information after a number (j) of single optimization mechanisms, and ε(o) means the probability of identifying the manufacturers' technical output information o∈O. In the continuous optimization mechanism, based on the actual technical input information (F,mc), the probability of the R&D institution's realizing the goal of benefit maximization is $K^{\Lambda }\left(T|F,mc\right)$. Since the R&D institution and the manufacturers have the same goal of benefit maximization, the probability of realizing their goal is equal, that is $K^{\Lambda }\left(T|F,mc\right)=\sum_{j\in J}\sum_{o\in O^{r}}k_{j}\varepsilon \left(o\right)$.

The probability of the other party's achieving the goal of benefit maximization is determined by considering the relationship between R&D institutions and manufacturers in the allocation of innovation factors and analyzing the identification and transformation process of technical information in the input and output processes. According to Bayesian principle, the proportion of the probability $k_{j}\varepsilon \left(o\right)$ of the manufacturers' realizing benefit maximization to the probability $K^{\Lambda }\left(T|F,mc\right)$ of the R&D institution's realizing benefit maximization refers to the probability determined by the R&D institution in terms of the manufacturers' realizing benefit maximization, which is stated as follows:

(7) $$\theta^{\Lambda }\left(o,j|T,F,mc\right)=k_{j}\varepsilon \left(o\right)/K^{\Lambda }\left(T|F,mc\right)$$

According to equation (7), where the R&D institution, based on the actual technical input information (F,mc) and its benefit maximization goal T, determines the probability of the manufacturers’ realizing benefit maximization when the technical output information is o after a number (j) of single optimization mechanisms, reflects the relationship between the two parties in the processes of identification and transformation of their technical information, and analyzes the correlation between the input and output of innovation factors.

#### 3.4.3 The impact of the simultaneous generation of actual technical input information and reported information on the benefit maximization

In the continuous optimization mechanism of IFAE Λ, the expected benefit at the time of simultaneous generation of R&D institution's actual technical input information and reported information $\left(F,mc,F^{r},mc^{r}\right)$ is $\Omega^{\Lambda }\left(T|F,mc,F^{r},mc^{r}\right)$. Due to the incomplete technical input information caused by subjective judgment, the expected benefit of the continuous optimization mechanism fluctuates greatly, which can be broken down into:

(8) $$\Omega^{\Lambda }\left(T|F,mc,F^{r},mc^{r}\right)=\sum_{j\in J}\sum_{o\in O}\theta^{\Lambda }\left(o,j|T,F^{r},mc^{r}\right)\Omega^{\lambda_{j}}\left(F^{r},mc^{r}|F,mc,o\right)$$

On the right side of equation (8), the simultaneous generation process is transformed into a sequential change process from conditions to results, and the impact of technical information changes on the benefit target is further analyzed, which is subdivided into two components. Firstly, after a number (j) of single optimization mechanisms λ, the R&D institution reports the technical input information $\left(F^{r},mc^{r}\right)$ and gets the benefit $\Omega^{\lambda_{j}}\left(F^{r},mc^{r}|F,mc,o\right)$. This benefit is conditional on the actual technical input information (F,mc) of the R&D institution and the actual technical output information o of the manufacturers. The R&D institution continuously reports more complete technical input information, and thus the expected benefit $\Omega^{\lambda_{j}}\left(F^{r},mc^{r}|F,mc,o\right)$ becomes higher, the way to achieve which is to continuously mine the technical input information through sequential identification. In the sequential change process of the actual technical input information and the reported information from conditions to results, the technical input information is fully identified, thus significantly improving the input-output efficiency. Secondly, the R&D institution determines that the probability of the manufacturers' achieving benefit maximization is $\theta^{\Lambda }\left(o,j|T,F,mc\right)$. When the R&D institution and the manufacturers' benefit maximization goal are consistent, and both parties report more complete technical information, the probability of each party's judgment of the other's realizing benefit maximization goal is higher. It can be seen that the continuous optimization mechanism is divided into a number (j) of single optimization mechanisms. Based on the actual technical input information, the R&D institution gradually reports technical input information during the allocation of innovation factors to obtain more information step by step. The single optimization mechanism helps gradually obtain more complete technical input information. The R&D institution can determine the probability of the manufacturers' achieving benefit maximization in each single optimization mechanism more accurately. Therefore, the correct information strategy selected by R&D institutions and manufacturers is to continuously report complete technical information.

#### 3.4.4 The impact of sequential generation of actual technical input information and reported information on benefit maximization

In the continuous optimization mechanism of IFAE Λ, the R&D institution, based on the actual technical input information (F,mc), reports the information $\left(F^{r},mc^{r}\right)$ and thus obtains the expected benefit $\Omega^{\Lambda }\left(F^{r},mc^{r}|F,mc\right)$. This includes two steps, which is used to analyze the constituent elements, influencing factors and benefit changes in the gradual transformation of incomplete technical information into complete technical information. After $\left(F^{r},mc^{r}\right)$ and (F,mc) are identified in two steps, they can also be compared with incomplete technical input information to obtain an accurate probability ε(o) of information identification and transformed shares $k_{j}$, dynamically reflecting interference factors in the change in technical information. $\left(F^{r},mc^{r}\right)$ and (F,mc) are generated step by step. After repeated absorptions and transformations, more technical input information is acquired. The expected benefit $\Omega^{\Lambda }\left(F^{r},mc^{r}|F,mc\right)$ can be subdivided into:

(9) $$\Omega^{\Lambda }\left(F^{r},mc^{r}|F,mc\right)\equiv \sum_{\Lambda \in \Lambda \left(F^{r},mc^{r}\right)}\theta^{\Lambda }\left(T|F^{r},mc^{r}\right)\max\left\{\Omega_{o}^{\Lambda }\left(T|F,mc,F^{r},mc^{r}\right),\alpha T\right\}+\sum_{j\in J}\sum_{o\in m_{0}\left(F^{r},mc^{r},o\right)}k_{j}\varepsilon \left(o\right)\Omega^{\lambda_{j}}\left(F^{r},mc^{r}|F,mc,o\right)$$

The first item on the right side of equation (9) refers to the random change in the expected benefit $\Omega_{o}^{\Lambda }\left(T|F,mc,F^{r},mc^{r}\right)$ of the R&D institution when the actual technical input information (F,mc) and the reported information $\left(F^{r},mc^{r}\right)$ are generated at the same time. The factors that can be found by continuous mining are obviously different from the incomplete technical input information share α. The deviation between the actual technical input information and the reported information includes both objective information differences and incomplete technical information formed by subjective knowledge. More complete technical input information can be obtained through repeated cognition, recognition, transformation and application. However, the incomplete technical input information share α is a primary error, which is difficult to be completely eliminated. The R&D institution reports technical input information $\left(F^{r},mc^{r}\right)$, which is not only the main way to obtain technical input information, but also the major basis for manufacturers to determine the completeness of technical input information.

The second item on the right side of the equation is the single optimization mechanism λ, in which the R&D institution, based on the actual technical input information (F,mc) and the manufacturers' actual technical output information o, reports that the benefit of the technical input information $\left(F^{r},mc^{r}\right)$ is $\Omega^{\lambda_{j}}\left(F^{r},mc^{r}|F,mc,o\right)$. The actual technical input information (F,mc) and reported information $\left(F^{r},mc^{r}\right)$ of the R&D institution are generated step by step to obtain more information. The probability of the manufacturers' achieving its benefit maximization goal is $k_{j}\varepsilon \left(o\right)$, and the corresponding expected benefit is $k_{j}\varepsilon \left(o\right)\Omega^{\lambda_{j}}\left(F^{r},mc^{r}|F,mc,o\right)$. After a number (j) of single optimization mechanisms, when the R&D institution reports complete technical input information, the manufacturers' share of technical achievements transformation will gradually increase, the probability of identifying technical information will continue to rise, and the contribution to the R&D institution's benefit will be significantly greater. When the single optimization mechanism has been operated for many times, the R&D institution gradually obtains more complete technical information after multiple recognitions and identifications of the actual technical input information, and then gives priority to reporting complete technical input information, thus realizing more accurate allocation of innovation factors and optimizing the input-output efficiency. As a result, hypothesis 5 is proposed as follows.

Hypothesis 5 The continuous optimization mechanism of IFAE enables manufacturers to obtain the expected benefit and helps the R&D institution to achieve its benefit maximization goal. The goal of the latter can be achieved only through the manufacturers' complete technical output information. The reporting of complete technical information by R&D institutions and manufacturers can not only enhance their benefits but also encourage the other party to provide complete technical information, which is conducive to the full absorption and transformation of technical information of both sides.

## 4 Industries, indicators and data processing

### 4.1 Selection of industries

In the empirical analysis, 73 listed companies in software development were selected as R&D institutions and 596 listed companies in processing and manufacturing were chosen as manufacturers, from Shanghai and Shenzhen stock markets of China. According to the industrial classification standards of Shenyin Wanguo, 596 processing and manufacturing companies are categorized into 5 different industries, namely 126 ones in electrical equipment, 143 ones in electronic components, 67 ones in auto parts, 57 ones in the communication equipment, and 203 ones in the pharmaceuticals & biotechnology.

### 4.2 Index acquisition and data processing

The data comes from the online data platform of Shanghai Securities News. The indicators for analysis are selected from the 2006–2018 financial statements of the chosen companies, mainly including three types.

Firstly, three factor input-related indicators contain material factor input, human capital input and intellectual capital input, which are represented separately by the changes in three financial indicators, namely, fixed assets, cash paid to and for employees, and intangible assets.

Secondly, two output benefit-related indicators reflect the output results of R&D institutions’ and manufacturers' innovation factor input, which are represented by earnings per share and operating revenues. The IFAE mainly reflects the proportional relationship between the innovation factor input of R&D institutions and the output of manufacturers after the absorption and transformation of technical information. In this empirical analysis, 73 listed companies in software development and 596 listed companies from 5 different processing and manufacturing industries are matched as input side and output side respectively. If it is difficult to achieve this between the given two companies, the IFAE is calculated through random matching. The descriptive statistical results and growth changes in main indicators are shown in Table 1.

**Table 1:** Descriptive statistics of main indicators for the information about the input and output of innovation factors.

Industry	Indicator Type	Fixed assets	Cash paid to and for employees	Intangible assets	Earnings per share	Operating revenues	Amortization of intangible assets	Net cash flow in operating activities	Net asset value per share	Accrued depreciation and amortization	Surplus reserves per share
		(1)	(2)	(3)	(4)	(5)	(6)	(7)	(8)	(9)	(10)
Software development industry	Average	22132.1	28980.46	9994.34	0.3857	35.9043	1096.586	−387.54	4.0633	3868.078	0.1632
	Standard deviation	35652.92	51781.57	27555.71	0.4509	305.1065	1892.948	14616.57	3.2098	6193.776	0.1316
	Average annual growth rate	14.10	22.33	32.98	−0.54	2.93	28.42	5.06	6.26	19.70	2.13
Electrical equipment industry	Average	108029.1	28856.8	20153.05	0.3592	18.65	1090.41	126.3613	3.9641	10184.54	0.1746
	Standard deviation	244498.8	69956.46	48713.62	0.5531	44.87	2640.588	3338.959	2.7687	22207.68	0.1595
	Average annual growth rate	19.94	15.77	17.55	−6.97	−2.84	23.39	15.49	4.48	19.76	0.2916
Electronic components industry	Average	133070.8	36407.24	17742.46	0.2935	44.6805	1334.098	97.908	3.6957	17577.26	0.1627
	Standard deviation	541971.6	87679.54	57476.7	0.5221	636.5589	5106.799	1611.341	2.5027	71541.81	0.1437
	Average annual growth rate	24.30	25.80	24.64	2.36	8.08	32.19	14.56	4.99	24.77	0.12
Auto parts industry	Average	120579.7	51904.22	31771.92	0.4654	18.2357	2237.973	63.1196	4.3058	18778.3	0.2649
	Standard deviation	270065.9	179187.8	165816.7	0.6073	54.1871	13263.94	1450.187	3.2271	57097.99	0.2987
	Average annual growth rate	17.31	26.01	22.73	0.34	1.09	33.73	−43.83	5.71	21.14	2.16
Communication equipment industry	Average	59342.84	43388.61	13543.15	0.3157	23.1862	1997.419	−9.9281	3.8129	10055.03	0.2036
	Standard deviation	139578.1	179551	50520.98	0.5328	89.4237	10677.59	2375.894	2.6017	29705.81	0.2269
	Average annual growth rate	15.23	15.65	28.03	−3.67	−2.29	22.28	−11.20	3.59	17.01	0.63
Pharmaceuticals & biotech industry	Average	74222.7	25015.54	19165.37	0.4709	21.1775	931.7495	52.8935	4.2734	7795.001	0.2584
	Standard deviation	111167.5	43949.27	38646.47	0.5964	77.2874	767.4139	3496.927	5.5733	11446.05	0.2974
	Average annual growth rate	13.81	19.40	16.41	4.68	−76.48	21.41	20.53	6.25	15.47	1.17

Notes: (1) The growth of indicators reflects the growth rate of the average value of 2007–2018 relative to that of 2006–2017; (2) the IFAE indicates the proportional relationship between the output of technical achievements of manufacturers and the input of R&D institutions for technical innovation, reflecting the input-output efficiency in the allocation of innovation factors.

Thirdly, technical information-related indicators are included. Since the annual report does not provide technical information-related indicators, technical information needs to be acquired by processing input and output data. See the notes to Table 2 for the corresponding treatment methods.

**Table 2:** Analysis of generation processes of technical information with different completeness levels.

Industry	Complete technical information	Relatively complete technical information	Incomplete technical information	Interaction terms for technical information			
				Incomplete technical input information*Incomplete technical output information	Incomplete technical input information*Complete technical output information	Complete technical input information*Complete technical output information	Complete technical input information*Complete technical output information
	(1)	(2)	(3)	(4)	(5)	(6)	(7)
Software development industry	4.3990	4.3990	4.7183				
Electrical equipment industry	5.7679	0.2708	0.1797	0.4753	0.5516	0.3421	0
Electronic components industry	0.6322	0.5143	0.9283	0.8127	0.5741	0.3659	0.6368
Auto parts industry	0.6573	0.2397	0.2671	0.8119	0.5328	0.4903	0.4364
Communication equipment industry	0.6101	0.3241	1.7158	1.8590	0.5922	0.7025	0.9977
Pharmaceuticals & biotech industry	0.6781	0.2651	0.1995	2.1779	0.5285	1.0407	0.2050

Notes: (1) The information on technical input reflects the year-on-year growth of intangible assets invested by 73 listed companies in software development. Due to the absence of introducing variables for weighting, complete information on technical input contains minimal interference information. In the case of relatively more complete information on technical input, two variables (the shares of cash flow in the net income from business activities, and net asset value per share) are introduced for weighting. In the case of incomplete information on technical input, apart from the above two variables, another two variables (calculated and extracted depreciation and amortization in the current period, and surplus reserves per share) are introduced for further weighting to reflect more incomplete information on technical input; (2) The information on technical output reflects the year-on-year growth of operating revenues of 596 listed companies, the processing method for the information on technical output in the three states is the same as that for information on technical input.

## 5 Analysis of empirical results

### 5.1 Analysis of the changes in benefits from innovation factor allocation in the case of technical information with different degrees of completeness and verification of the results

The columns (1)–(3) and (4)–(6) in Table 3 reflect the changes in the contribution of the input of different factors to earnings per share when the incomplete technical input information of the software development industry and the incomplete technical output information of five processing and manufacturing industries are gradually transformed into relatively complete technical input information and relatively complete technical output information.

**Table 3:** Analysis of the changes in benefits from the innovation factor allocation in the case of incomplete technical information, and verification of results.

Industry	OLS			Material factor investment	Human capital investment	Intellectual capital investment	Correlation test	Exogeneity test	Test of the endogeneity of explanatory variables	Parameter constraint test
	Material factor investment	Human capital investment	Intellectual capital investment							
	(1)	(2)	(3)	(4)	(5)	(6)	(7)	(8)	(9)	(10)
Software development industry	0.8022	0.0142	−0.0019	−0.0723	0.7884	0.6699	2.5604	1.4447	17.4534	8.49
	(0.0011)	(0.0075)	(0.0010)	(0.0307)	(0.4083)	(0.4737)	0.0783	0.2294	0	0.0753
Electrical equipment industry	0.2106	0.1823	0.0024	0.0198	0.3031	0.0063	1.6971	3.6896	10.6276	6.69
	(0.0063)	(0.0327)	(0.0023)	(0.0205)	(0.2078)	(0.0175)	0.1837	0.0548	0.0012	0.1573
Electronic components industry	0.1054	0.1916	−0.0048	−0.0475	1.1730	0.0916	0.6651	0.1268	7.7679	3.11
	(0.0015)	(0.0300)	(0.0010)	(0.0661)	(1.1885)	(0.0602)	0.0514	0.1722	0.0054	0.5388
Auto parts industry	0.3260	0.1858	−0.0090	0.2532	0.4564	0.0156	1.8789	0.4479	2.5695	6.38
	(0.0384)	(0.0564)	(0.0048)	(0.8799)	(3.3062)	(0.0770)	0.1538	0.2039	0.1096	0.1724
Communication equipment industry	0.2171	0.2934	−0.0052	0.0312	1.2387	0.0214	0.6112	0.0082	17.8384	2.79
	(0.0178)	(0.0483)	(0.0026)	(0.0462)	(1.7443)	(0.0290)	0.0543	0.1928	0	0.5933
Pharmaceuticals & biotech industry	0.0007	−0.0010	−0.0004	−0.0006	0.0310	0.0203	0.6950	0.6071	15.4416	89.01
	(0.0093)	(0.0024)	(0.0005)	(0.0106)	(0.0374)	(0.0000)	0.0499	0.4359	0.0001	0

Notes: (1) In OLS regression analysis, the dependent variable for the six industries is the changes in earnings per share, and the independent variables include three indicators, i.e. fixed assets, cash paid to and for employees, and amortization of intangible assets. In particular, the independent variables introduced for the software development industry include incomplete technical input information, and the independent variables introduced for the five processing and manufacturing industries include incomplete technical output information; (2) In the regression analysis of relatively complete technical information, the disturbance term contains many unmeasurable factors, which makes it impossible to test the correlation between the explanatory variables and the disturbance term. Hence, more effective instrumental variables can be introduced to gradually transform the incomplete technical information into relatively complete technical information and further complete technical information; (3) In GMM regression analysis, the endogenous explanatory variable for software development industry is relatively complete technical input information, the endogenous explanatory variables for the other five processing and manufacturing industries are relatively complete technical output information, and the instrumental variables for the six industries refer to the share of net incomes from changes of the value in total profits and the share of non-recurring gains and losses in net profits; (4) The correlation test of instrumental variables is to determine the correlation between instrumental variables and endogenous explanatory variables, which is expressed by the regression statistics and critical values in the first stage. The exogeneity-purpose test uses the over-identification test method. The original hypothesis is "H0: all instrumental variables are exogenous"; (5) The endogeneity-purpose test of endogenous explanatory variables uses the Hausman test method, and the original hypothesis is "H0: endogenous explanatory variables are exogenous"; (6) The parameter constraint test is to identify the characteristics of differences in regression coefficients of two different methods.

The empirical results show that the contribution of material factors of six industries to the growth of earnings per share has decreased to a certain extent. While the contributions of human capital and intellectual capital investments to the growth of earnings per share show an upward trend. When incomplete technical information is transformed into relatively complete technical information, the six industries obtain more sufficient technical information, the technical achievements in the forms of knowledge and information are effectively utilized, and the role of knowledge-form technical information in benefit growth is more significant. The growth of earnings per share is increasingly relying on the improvement of workers' knowledge and skills and more complete technical information, rather than the consumption of material factors. Relatively complete technical information has played a more significant role in optimizing the structure of factor input and improving the rate of return on factors. Therefore, hypothesis 2 is verified.

Columns (7) and (8) in Table 3 refer to the results of verifying the validity of instrumental variables by GMM regression analysis. According to the verification results concerning the correlation between two instrumental variables of relatively complete technical information and endogenous explanatory variables, the hypothesis about the correlation in terms of electrical equipment industry and auto parts industry cannot be rejected at the 10 % significance level. At the 5 % significance level, the hypothesis regarding the correlation in terms of software development industry, electronic component industry and communication equipment industry cannot be rejected, which meets the requirements for the correlation between instrumental variables and endogenous explanatory variables. At the 1 % significance level, the hypothesis regarding the correlation in terms of pharmaceuticals and biotech industry cannot be rejected, and the significance level of this industry is relatively low. The results of testing the exogeneity of instrumental variables show that only the hypothesis concerning the exogeneity in the case of electrical equipment industry is rejected at the 10 % significance level, and the hypotheses concerning the exogeneity in terms of the other five industries cannot be rejected at the 10 % significance level. The exogenous characteristics of instrumental variables are significant, which is not related to the errors from regression, so that the regression results reflect more changes in technical information.

Column (9) in Table 3 lists the results of verifying the endogeneity of explanatory variables based on Hausman method in the case of relatively complete technical information. According to the results, the hypothesis regarding the exogeneity of explanatory variables cannot be rejected for the auto parts industry at the 10 % significance level, indicating that the endogenous characteristics of explanatory variables are not obvious. The hypotheses regarding the exogeneity in terms of the other five industries are rejected at the 1 % significance level, revealing that the explanatory variables of the five industries have some endogenous characteristics. Column (10) refers to the results of parameter constraint testing in the case of relatively complete technical information. According to the results, the hypothesis that there are differences between the two regression coefficients in terms of the pharmaceuticals & biotech industry is rejected at the 1 % significance level. The unobservable factors in relatively complete technical information have no significant impact. The technology intensity of the pharmaceuticals & biotech industry is relatively high. Technical achievements in the forms of knowledge and information are abundant. The path for transformation and application is shorter, and the difference between sample companies is relatively smaller. Accordingly, the hypothesis that there are differences between the two regression coefficients in terms of the other five industries cannot be rejected at the 5 % significance level. There are statistical differences between the coefficients from the two regression methods, which can explain the internal technical and economic correlations between variables.

Different from Columns (4)–(6) in Table 3 listing data concerning relatively complete technical information, Columns (1)–(3) in Table 4 show that when the software development industry and the five processing and manufacturing industries obtain complete technical information, the contribution of factors changes more significantly. Under the condition of complete technical information of the six industries, the contribution of material factor investment to the growth of earnings per share is declining, while the contribution of human capital and intellectual capital investment to the growth of earnings per share shows an upward trend. The realization of complete technical information is the main way to continuously enhance the benefits from the allocation of innovation factors. It can improve the allocation of innovation factors more efficiently, and remarkably increase the benefits of R&D institutions and manufacturers without investing more innovation factors.

**Table 4:** Empirical analysis of the influence on the earnings from technical innovation in the case of complete technical information, and verification of results.

Industry	GMM(Complete technical information)			Test for instrumental variables		Test for the endogeneity of explanatory variables	Parameter test	Equivalence test		
	Material factor investment	Human capital investment	Intellectual capital investment	Correlation test	Exogeneity test			Material factor investment	Human capital investment	Intellectual capital investment
	(1)	(2)	(3)	(4)	(5)	(6)	(7)	(8)	(9)	(10)
Software development industry	−0.0722	0.7244	0.5433	1.4964	5.2957	44.06	13.59	9.14	35.71	0.172
	(0.0259)	(0.3451)	(0.3908)	0.202	0.1514	0	0.0087	0.003	0	0.678
Electrical equipment industry	−0.0373	1.4105	0.7122	3.9356	16.646	64.8458	22.5	29.31	109.95	16.074
	(0.0140)	(0.1858)	(0.0046)	0.0635	0.5208	0	0.0002	0	0	0
Electronic components industry	−0.1314	1.7442	0.116	1.7894	10.96	78.69	17.53	2.6037	25.89	0.0063
	(0.0798)	(0.2225)	(0.0080)	0.7286	0.7119	0	0.0015	0.107	0	0.937
Auto parts industry	−0.396	2.9772	0.064	6.1832	6.1714	25.78	15.28	0.0664	22.45	0.4083
	(0.2964)	(1.2152)	(0.0667)	0.0701	0.6036	0	0.0042	0.797	0	0.523
Communication equipment industry	−0.1146	1.9894	0.0846	1.2615	8.2517	34.27	21.59	3.0818	24.17	3.1501
	(0.2068)	(1.6622)	(0.0474)	0.2843	0.6411	0	0.0002	0.079	0	0.076
Pharmaceuticals & biotech industry	−0.0296	0.1413	0.0311	2.0663	0.7706	0.1097	5.15	1.6985	47.39	4.1833
	(0.0036)	(0.1599)	(0.0003)	0.0828	0.58	0.0441	0.0272	0.192	0	0.041

Note: In the GMM regression analysis, the endogenous explanatory variable for the software development industry is complete technical input information, the endogenous explanatory variables for the other five processing and manufacturing industries are complete technical output information, and apart from the instrumental variables listed in Table 3, another two instrumental variables for the six industries are introduced, including the provisions for asset impairment and the calculation and extraction of depreciation and amortization of the sample companies, so that the endogenous explanatory variables reflect more changes in technical information.

Columns (4)–(6) in Table 4 describe the results of testing the validity of instrumental variables and endogeneity of explanatory variables in the case of complete technical information. The hypothesis regarding the correlation between instrument variables and endogenous explanatory variables for electrical equipment industry, auto parts industry, and pharmaceuticals & biotech industry cannot be rejected at the 5 % significance level. On the basis of relatively complete technical information, another two instrument variables (provisions for asset impairment, and calculation and extraction of depreciation and amortization) are introduced to more comprehensively reflect the changes in technical information. The four instrumental variables have a certain degree of correlation with endogenous explanatory variables, and the information experiences a small loss and better reflects the technical and economic correlations, so as to help obtain an ideal regression effect. The hypothesis concerning the correlation between instrumental variables and endogenous explanatory variables for software development industry, electronic component industry, and communication equipment industry cannot be rejected at the 10 % significance level. This indicates that variables are significantly correlated, knowledge-form technical information is fully transmitted, and the regression results are more consistent with the technical and economic correlations between variables. The test results also show that the hypothesis about the exogeneity of instrumental variables for the six industries cannot be rejected at the 10 % significance level. This means that the instrumental variables have a significant mitigating effect on the disturbance term. The regression results reflect the technical and economic correlations more comprehensively, and complete technical information plays a more significant role. The test for endogeneity of explanatory variables uses the Hausman method. The hypothesis with respect to the exogeneity of explanatory variables for the six industries is rejected at the 5 % significance level. The endogenous characteristics are obvious, and the regression results are strongly explained.

Column (7) in Table 4 shows the results of the parameter constraint test in the case of complete technical information. The hypothesis that there are differences between the two regression coefficients for all the industries is rejected at the 5 % significance level. By introducing four instrumental variables, complete technical information for six industries is acquired, which can help completely and accurately reflect the changes in cutting-edge technologies of each sample company within an industry and make the input-output ratio closer to an optimal state. First of all, with regard to the test for the relationship between variables in the same period, there is a significant difference between the regression results acquired by using instrumental variables in GMM analysis and those obtained by using the OLS method. The differences between the coefficients of individual variables can be effectively lessened by introducing effective instrumental variables. Secondly, the given instrumental variables are not completely related to the endogenous explanatory variables. They do not absorb all the interference factors in the relatively complete technical information, and ignore a large amount of information on nonlinear changes. There is still residual in the estimation results. Finally, the regression method for instrumental variables can be further improved by introducing more effective instrumental variables to ensure that instrumental variables truly and objectively reveal the changes in actual technical information, reflect the characteristics of staged technical progress, including information such as value compensation and changes of incomes, and reduce regression bias.

Columns (8)–(10) in Table 4 list the results of equivalence testing in terms of material factors, human capital and intellectual capital, which are used to verify whether the regression coefficients have equivalence characteristics in the case of replacing two instrumental variables with four instrumental variables and introducing complete technical information. According to the results of equivalence testing for material factor input, the hypothesis concerning the equivalence of regression coefficients in the case of software development industry and electrical equipment industry is rejected at the 1 % significance level. Complete technical information has a significant impact on regression coefficients, causing a large difference in regression coefficients and a significant change in the factor allocation proportion. The hypothesis regarding the equivalence of regression coefficients in the case of communication equipment industry is rejected at the 10 % significance level. Affected by complete technical information, there is a trend of differential changes in regression coefficients, and the change in the structure of material factor investments leads to a greater fluctuation of earnings from innovation. The hypothesis about the equivalence of regression coefficients in the case of electronic components industry, auto parts industry and pharmaceuticals & biotech industry cannot be rejected at the 10 % significance level, indicating that complete technical information has no obvious effect on optimizing the proportion of factor allocation and fails to bring significant structural changes in the material factor investment. According to the results of equivalence testing for human capital investment, the hypothesis with respect to the equivalence of regression coefficients for six industries is rejected at the 1 % significance level. Complete technical information makes the structure of human capital investment change significantly, quickly adapt to changes in technical information, possess strong self-regulation characteristics, and have a high correlation with changes in technical information. According to the results of equivalence testing for intellectual capital investment, the hypothesis about the equivalence of regression coefficients in the case of software development industry, electronic component industry and auto parts industry cannot be rejected at the 10 % significance level. Complete technical information does not lead to differentiated changes in the coefficient of intellectual capital investment. The speed of change in technical information is relatively higher. The intellectual capital investment, affected by factors such as updating and replacement, has a relatively slower cycle. The hypothesis regarding the equivalence of regression coefficients in the case of communication equipment industry cannot be rejected at the 5 % significance level, while the hypothesis concerning the equivalence of regression coefficients in the case of electrical equipment industry and pharmaceuticals & biotech industry is rejected at the 5 % significance level. The intellectual capital investment is realized mainly through information transmission, path diffusion and other ways. Only when it penetrates into physical-form elements can it make a contribution to earnings. It is closely related to the degree of correlation between the input and output of the industry to which it belongs. Due to technical information-related factors, there is a great difference in intellectual capital investment of different industries. Therefore, hypothesis 2 is verified.

### 5.2 Testing for the optimization mechanism of IFAE in the case of incomplete technical information

#### 5.2.1 Testing for the exogeneity of instrumental variables

According to the results of Column 1 in Table 5, the test for the exogeneity of instrumental variables for 6 industries fails to reject the hypothesis at the 10 % significance level, which meets the exogeneity requirements of instrumental variables. The instrumental variables can explain disturbance factors in the regression more significantly, and the regression results reflect the technical and economic correlations between variables more accurately. The results of testing the validity of instrumental variables show that the interaction between the technical information of the R&D institution and that of manufacturers can accurately reflect the absorption and transformation of technical information in the allocation of innovation factors. Instrumental variables are effective, and the change in technical information plays a more significant role in the optimization mechanism of IFAE.

**Table 5:** Changes in IFAE and analysis of the test results of optimization mechanisms.

Industry	Exogeneity test	Changes in efficiency of innovation factor allocation		Three single optimization mechanisms				Continuous optimization mechanism	
		Incomplete technical information	Complete technical information	Ⅰ:Incomplete technical output information*Incomplete technical input information, Incomplete technical output information*Complete technical input information	Ⅱ:Incomplete technical input information*Incomplete technical output information, Incomplete technical input information*Complete technical output information	Ⅲ:Incomplete technical output information* Incomplete technical input information, Incomplete technical output information* Complete technical input information, Incomplete technical input information* Complete technical output information	Test for mean value	Incomplete technical input information*Incomplete technical output information, Complete technical input information*Complete technical output information	Test for mean value
	(1)	(2)	(3)	(4)	(5)	(6)	(7)	(8)	(9)
Software development industry	1.7877	0.3904	0.0044	2.5871	1.9074	2.0814	92.54	2.4181	18.37
	0.1812			0.0724	0.1157	0.0836	0	0.1052	0
Electrical equipment industry	1.3625	0.1573	0.0207	11.88	27.07	18	105.35	15.72	246.95
	0.2431			0.0604	0.1629	0.0931	0	0.1158	0
Electronic components industry	0.1604	0.4646	0.0049	1.5044	4.6849	1.0282	243.89	3.1704	2.0492
	0.6888			0.0426	0.0292	0.1287	0	0.0128	0.1532
Auto parts industry	0.4716	0.3147	0.0098	3.9843	5.1518	1.3072	97.26	2.1742	11.17
	0.4922			0.6508	0.5072	0.1928	0	0.2039	0.0009
Communication equipment industry	2.3174	0.2936	0.0063	1.6607	1.0887	1.1752	63.39	2.1753	27.75
	0.1279			0.1058	0.2057	0.1049	0	0.0906	0
Pharmaceuticals & biotech industry	0.0493	0.0669	0.0287	1.2186	2.8407	2.0369	429.12	1.4705	317.36
	0.8243			0.1074	0.0920	0.1753	0	0.1193	0

Notes: (1) Empirical analysis is based on GMM method. The dependent variable is IFAEjt, the independent variable refers to the information on changes in the investment of material factors, human capital and intellectual capital, and the endogenous explanatory variable is an interaction term for complete technical input information and complete technical output information; (2) The test for exogeneity adopts an over-identification method to determine whether the instrumental variables have exogenous characteristics.

#### 5.2.2 Testing for the validation of instrumental variables

Column 4 in Table 5 refers to the single optimization mechanism I. The instrumental variables include two interaction terms concerning the interaction of incomplete technical output information of processing and manufacturing industry with incomplete technical input information and complete technical input information of software development industry respectively. As the results show, the test fails to reject the hypothesis regarding the correlation between endogenous explanatory variables and instrumental variables for electronic component industry at the 1 % significance level, for software development industry and electrical equipment industry at the 5 % significance level, and for automobile parts industry, communication equipment industry and pharmaceuticals & biotech industry at the 10 % significance level. For the six industries, their instrumental variables all include incomplete technical output information. As the incomplete technical input information of the R&D institution gradually becomes complete information, there is a closer correlation between technical information of different industries, a larger amount of effective information, and a more significant correlation between instrumental variables and endogenous explanatory variables. When the R&D institution provides sufficient technical input information, there are abundant reusable technical achievements in the form of knowledge and information in the allocation of innovation factors, which can significantly reduce the input costs of the R&D institution without increasing the output level, thus improving the IFAE.

Column 5 in Table 5 refers to the single optimization mechanism II. The instrumental variables include two interaction terms regarding the interaction of the R&D institution's incomplete technical input information and the processing with the manufacturing industry's incomplete technical output information and complete technical output information respectively. In terms of the processing and manufacturing industry, more complete technical input information is recognized and identified in the allocation of innovation factors, leading to gradual acquisition of sufficient technical output information and deriving a large amount of new products and new achievements. The instrumental variables contain a large amount of valuable technical information. The test fails to reject the hypothesis concerning the correlation between endogenous explanatory variables and instrumental variables for electronic component industry and pharmaceuticals & biotech industry at the 1 % and 5 % significance levels, and fails to reject the hypothesis concerning the correlation between endogenous explanatory variables and instrumental variables for the other four industries at the 10 % significance level. The test results show a significant correlation between instrumental variables and endogenous explanatory variables. In the allocation of innovation factors, more incomplete technical output information is transformed into complete technical output information, providing more information for processing and manufacturing industry and contributing to product categories, faster upgrading of new products and effective release of potential capacity. In the case of no more costs of innovation input by software development industry, complete technical output information is conducive to improving the allocation of innovation factors, and further enhance the output level of processing and manufacturing industry.

Column 6 in Table 5 refers to the single optimization mechanism Ⅲ. The instrumental variables include two interaction terms regarding the interaction of incomplete technical output information of the processing and manufacturing industry with incomplete technical input information and complete technical input information of the R&D institution, and one interaction term for incomplete technical input information and complete technical output information. The test fails to reject the hypothesis concerning the correlation between endogenous explanatory variables and instrumental variables for software development industry and electrical equipment industry at the 5 % significance level, and for the other four industries at the 10 % significance level. As long as the R&D institution provides more technical input information, the innovation input will decrease without reducing the output level of manufacturers, and technical achievements will be fully utilized. Moreover, when manufacturers provide more technical output information, innovation output will increase by deeply tapping the production potential of existing factors in the case of no more input of innovation factors.

Column 8 in Table 5 refers to the continuous optimization mechanism. The instrumental variable is an interaction term for incomplete technical input information of the R&D institution and incomplete technical output information of manufacturers. The regression coefficient for its relation with endogenous explanatory variables includes all technical information. The test fails to reject the hypothesis concerning the correlation between endogenous explanatory variables and instrumental variables for electronic component industry at the 1 % significance level, for communication equipment industry at the 5 % significance level, and for the other four industries at the 10 % significance level. The results of correlation tests for the six industries are quite different. There is a great difference in technology intensity of such industries, indicating a significant variance in their dependence on technical information. Both parties involved in the allocation of innovation factors obtain complete technical input information and complete technical output information. Any improvement in the allocation of innovation factors is realized in the case of no reduction in the innovation input costs of the R&D institution and no increase in the output level of any manufacturer, and the efficiency of the allocation of innovation factors is optimized. Therefore, hypothesis 3 is verified.

#### 5.2.3 Analysis of changes in IFAE caused by technical information with different levels of completeness

The regression analysis explores the shares of the disturbance term of IFAE in the expected value of IFAE in the case of complete technical information and incomplete technical information respectively, and analyzes the fluctuation range of IFAE brought by incomplete technical information. According to the results of Columns 2 and 3 in Table 5, for the six industries, the shares of disturbance terms in the expected value of IFAE in the case of complete technical information are respectively 0.0044, 0.0207, 0.0049, 0.0098, 0.0063 and 0.0287, significantly smaller than those in the case of incomplete technical information, which are respectively 0.3904, 0.1573, 0.4646, 0.3147, 0.2936 and 0.0669. Complete technical information leads to a stable change in IFAE, without significant fluctuations. Incomplete technical information contains more interference factors, which results in a mismatched input-output relationship in innovation factor allocation. The IFAE deviates from the optimal level, with great fluctuations.

#### 5.2.4 The mean values of technical information with different levels of completeness reflect the difference between endogenous explanatory variables and instrumental variables

Tests have been carried out to explore the degree of differentiation between endogenous explanatory variables and corresponding instrumental variables in the case of single optimization mechanism and continuous optimization mechanism. Results are listed in Columns 7 and 9 of Table 5. The original hypothesis is that all mean values are the same, that is, there is no difference between endogenous explanatory variables and instrumental variables. As the results show, the test fails to reject the original hypothesis only in the continuous optimization mechanism for the electronic component industry at the 10 % significance level, and their mean values are the same, with no significant difference. The mean values in the single optimization mechanism and the continuous optimization mechanism for the IFAE of the other five industries are all 0, which rejects the original hypothesis, that is, there is a significant difference in mean value. The regression analysis of instrumental variables and the endogenous explanatory variables can reflect the characteristics of technical and economic changes, which is a precondition for regression analysis of different types of technical information and IFAE. Thus, hypothesis 1 is verified.

### 5.3 Regression results of IFAE

In the regression analysis of IFAE, the endogenous explanatory variable refers to an interaction term for complete technical input information of the R&D institution and complete technical output information of manufacturers. In terms of three single optimization mechanisms and one continuous optimization mechanism for six industries, due to different instrumental variables, the endogenous explanatory variable brings differentiated contributions to the change in the IFAE. The regression results from Column 1 to Column 4 in Table 6 show that with respect to the software development industry, the electrical equipment industry and the auto parts industry, by using the GMM method, in three single optimization mechanisms and one continuous optimization mechanism, under the influence of different instrumental variables, the shares of the contribution of endogenous explanatory variables to the change in IFAE are respectively 0.4926, 0.8519, 0.4483, 0.7281, 0.5274, 0.9020, 0.8305, 0.8178, 0.0980, 0.8597, 0.4926 and 0.7704.

**Table 6:** Regression analysis of optimization mechanisms of IFAE based on technical information with different levels of completeness, and verification of results.

Industry	Efficiency of innovation factor allocation				Difference analysis of IFAE			
	Single optimization mechanisms			Continuous optimization mechanism	Single optimization mechanisms			Continuous optimization mechanism
	Ⅰ	Ⅱ	Ⅲ		Chi2(1)/P	Chi2(1)/P	Chi2(1)/P	Chi2(1)/P
	(1)	(2)	(3)	(4)	(5)	(6)	(7)	(8)
Software development industry	0.4926	0.8519	0.4483	0.7281	0.7361	1.946	2.7194	2.7103
	(0.7108)	(0.1004)	(0.2817)	(0.3902)	0.0274	0.4519	0.0272	0.0429
Electrical equipment industry	0.5274	0.9020	0.8305	0.8178	0.1539	3.0291	1.9017	0.0071
	(0.4427)	(0.4935)	(0.3408)	(0.4416)	0.0499	0.0321	0.0168	0.0382
Electronic components industry	0.7687	2.8334	1.7283	2.4905	0.1629	0.6072	0.4918	0.5208
	(1.0005)	(3.1281)	(2.0519)	(2.7318)	0.0691	0.0508	0.7054	0.0519
Auto parts industry	0.0980	0.8597	0.4926	0.7704	6.1706	5.6817	7.2806	6.0412
	(0.4968)	(0.7273)	(0.5349)	(0.6072)	0.113	0.6172	0.0129	0.6191
Communication equipment industry	−0.3534	−1.5985	−0.9281	−1.2074	2.1319	2.3716	2.9518	2.2619
	(0.4323)	(1.6721)	(0.8164)	(0.9175)	0.0596	0.0627	0.0758	0.0791
Pharmaceuticals & biotech industry	0.0899	4.5459	2.4118	3.9206	0.3671	0.3409	0.4121	0.3927
	(0.4985)	(4.5522)	(2.4910)	(3.7461)	0.0547	0.086	0.0601	0.0512

Notes: The IFAEjt-1 uses GMM regression method, and the independent variables are material factors, human capital and intellectual capital investments. One interaction term for complete technical input information and complete technical output information is adopted as an endogenous explanatory variable. The instrumental variables in the three single optimization mechanisms and continuous optimization mechanisms are the same as those in Table 5.

According to the results of Hausman test in Columns 5–8 of Table 6, the test fails to reject the hypothesis concerning the inexistence of a systematic difference in regression coefficient at the 1 % significance level. The difference in GMM regression coefficient is caused by different types of technical information, reflecting differential technical and economic relations between variables. The contributions of endogenous explanatory variables of the electronic component industry and pharmaceuticals & biotech industry to the changes in IFAE are respectively 0.7687, 2.8334, 1.7283, 2.4905, 0.0899, 4.5459, 2.4118 and 3.9206. The Hausman test fails to reject the hypothesis regarding a lack of a systematic difference in regression coefficients for the two industries at the 5 % significance level. Different instrumental variables have an unsystematic differentiated impact on the IFAE. The regression results are highly representative and can explain the internal relationship between variables. The contributions of endogenous explanatory variables of the communication equipment industry to the changes in IFAE are −0.3534, −1.5985, −0.9281 and −1.2074. The contributions of instrumental variables and endogenous explanatory variables to the changes in IFAE are negative. The Hausman test fails to reject the hypothesis about a lack of a systematic difference in regression coefficient at the 5 % significance level. This is because instrumental variables and endogenous explanatory variables well explain the changes in IFAE brought about by different types of technical information, which accurately reflects the relationship between them in differential changes and is consistent with the trend of changes in IFAE.

### *5.4* Quantile regression of technical information with different levels of completeness and IFAE

In the single optimization mechanism, there are three independent variables reflecting different types of technical information in turn. Two of them refer to interactions of the R&D institution's incomplete technical input information with the manufacturers' incomplete technical output information and complete technical output information, and another one refers to the interaction of the R&D institution's complete technical input information with the manufacturers' incomplete technical output information. Quantile regression was carried out for the three interaction terms and IFAE at the same time. The three quantiles were 0.1, 0.5 and 0.9. According to the quantile regression results from Column 1 to Column 9 in Table 7, in three single optimization mechanisms, the contributions of the three interaction terms as independent variables to changes in IFAE for the electrical equipment industry are respectively 0.0003, −0.0377, 2.8150, −0.0002, 0.5336, 3.7333, 0.0019, 0.2190 and 0.0356, showing three trends of changing, namely, a V shape, a continuous rise, and an inverted V shape. In the single optimization mechanisms I and II for the electronic component industry and auto parts industry, the contribution of incomplete technical information-related interaction terms to IFAE are respectively −0.0006, 0.0017, 2.3132, 0.0006, 0.6949, 4.6925, 0.0154, 0.5069, 1.2327, 0.2019, 0.3275 and 5.1846 at three quantiles, showing a continuous upward trend. In the single optimization mechanism III, the contributions of incomplete technical information in the electronic component industry to the IFAE are 0.0023,0.0319 and −0.6494, showing an inverted V-shaped trend of change. The contributions in terms of the auto parts industry are 0.0003, −0.0858 and −0.5441, indicating a continuous downward trend of change. In the single optimization mechanism I for the software development industry and communication equipment industry, such contributions are 0.0729, 0.0184, −0.6018, −0.0041, −0.0346 and −0.2023, presenting a continuous downward trend, and in the single optimization mechanisms II and III, such contributions are −0.0517, 0.1728, 1.9173, 0.0491, 0.5921, 2.4372, 0.0002, 0.2721, 2.2926, 0.0214, 0.0895 and 0.1956, showing a continuous upward trend. According to the quantile regression results from Column 10 to Column 12 in Table 7, in the continuous optimization mechanism for the above five industries, the contributions of incomplete technical information to the IFAE are respectively 0.0382, 0.4193, 2.0427, 0.0025, 0.1709, 3.3436, 0.0003, 0.8207, 7.8507, 0.2311, 0.7809, 6.1769, 0.0019, 0.3441 and 3.0606. The contributions of the software development industry and the four processing and manufacturing industries at the three quantiles have shown a continuous upward trend, which is formed on the basis of three single optimization mechanisms and enables limited technical information to be fully absorbed and transformed on the basis of each single optimization mechanism. Therefore, this helps avoid errors and deviation and guide the IFAE to achieve the optimal state to further play a significant role. With regard to the pharmaceuticals & biotech industry, due to its high-level specialization, highly intensive knowledge and information, and a relatively low proportion of processing and manufacturing, no regression results at the 0.1 quantile have been found in the quantitative analysis, so its analysis was ignored.

**Table 7:** Quantile regression analysis of optimization mechanisms of IFAE and different types of technical information.

Industry	single optimization mechanisms									Continuous optimization mechanism			Regression of the growth of net profit and disturbance term	Regression of the growth of cash flow per share and disturbance term
	Ⅰ			Ⅱ			Ⅲ			IIIO				
	Q1	Q2	Q3	Q1	Q2	Q3	Q1	Q2	Q3	Q1	Q2	Q3		
	(1)	(2)	(3)	(4)	(5)	(6)	(7)	(8)	(9)	(10)	(11)	(12)	(13)	(14)
Software development industry	0.0729	0.0184	−0.6018	−0.0517	0.1728	1.9173	0.0491	0.5921	2.4372	0.0382	0.4193	2.0427	−0.6893	−2.1001
	(0.0273)	(0.1840)	(1.5920)	(0.1921)	(0.4715)	(1.0713)	(0.1052)	(0.4715)	(0.9238)	(0.0914)	(0.6290)	(1.0471)	(0.6209)	(2.2448)
Electrical equipment industry	0.0003	−0.0377	2.815	−0.0002	0.5336	3.7333	0.0019	0.219	0.0356	0.0025	0.1709	3.3436	0.2337	0.494
	(0.0163)	(0.1292)	(2.0287)	(0.0033)	(0.1561)	(0.6999)	(0.0203)	(0.0939)	(0.9975)	(0.0024)	(0.1781)	(1.5456)	(0.8087)	(4.1382)
Electronic components industry	−0.0006	0.0017	2.3182	0.0006	0.6949	4.6925	0.0023	0.0319	−0.6494	0.000.	0.8207	7.8507	0.1748	−0.6869
	(0.0030)	(0.1084)	(1.2836)	(0.1084)	(0.1204)	(1.0122)	(0.0081)	(0.0858)	(0.5502)	(0.0033)	(0.1325)	(1.5164)	(1.6115)	(1.0497)
Auto parts industry	0.0154	0.5069	1.2327	0.2019	0.3275	5.1846	0.0003	−0.0858	−0.5441	0.2311	0.7809	6.1769		
	(0.1078)	(0.2793)	(2.4609)	(0.0637)	(0.3322)	(5.1718)	(0.0399)	(0.0578)	(0.3357)	(0.0690)	(0.3352)	(3.7551)		
Communication equipment industry	−0.0041	−0.0346	−0.2023	0.0002	0.2721	2.2926	0.0214	0.0895	0.1956	0.0019	0.3441	3.0606	1.9525	1.1919
	(0.0114)	(0.0517)	(0.1922)	(0.0151)	(0.2135)	(1.1538)	(0.0155)	(0.0721)	(0.2965)	(0.0097)	(0.2751)	(1.7848)	(0.6209)	(2.2448)
Pharmaceuticals & biotech industry		0.345	2.6686		0.329	2.369		−0.0273	−0.0815		0.3675	2.9645		
		(0.2616)	(2.0192)		(0.0968)	(0.9965)		(0.0112)	(0.1555)		(0.1040)	(1.1752)		

In particular, in the continuous optimization mechanism of IFAE, a regression analysis of the relationship of the regression disturbance term with the growth of the industries’ net profit and cash flow per share respectively. As the results in Columns 13 and 14 of Table 7 show, for the software development industry, a unit of regression disturbance term has a negative impact on the growth of net profit and cash flow per share, and the degrees of impact are −0.6893 and −2.1001 respectively. With respect to the electric equipment industry and the communication equipment industry, a unit of regression disturbance term has a positive impact on the growth of net profit and cash flow per share, and the degrees of impact are 0.2337, 1.9525, 0.4940 and 1.1919 respectively. Regarding the electronic component industry, a unit of regression disturbance term has a small impact on the growth of net profit, the degree of which is only 0.1748, while it has a negative impact on the growth of cash flow per share, the degree of which is −0.6869. The influence is produced mainly via incomplete technical information and the regression disturbance term for IFAE. The degree and orientation of influence depend on different industries. There is a significant difference between various industries. To obtain complete technical information, it is necessary to continue to perceive, recognize and absorb the information on original achievements in the processes of R&D, testing and manufacturing. Therefore, hypothesis 5 is verified.

### 5.5 Optimization mechanisms of IFAE in the case of technical information with different levels of completeness, and equilibrium analysis

Table 8 shows the relationship between the IFAE and the interaction terms of R&D institutions’ and manufacturers' technical information. In the regression analysis, the dependent variable is the IFAE. The observable variables in the single optimization mechanism include three interaction terms: the interactions of incomplete technical input information of the R&D institution with incomplete technical output information and complete technical output information of the manufacturers, and the interaction between complete technical input information of the R&D institution and the incomplete technical output information of the manufacturers. The linear term and quadratic term for the three observable variables are selected as independent variables of the regression analysis. The above three interaction terms represent changes in incomplete technical information during the allocation of innovation factors. At least one of the R&D institutions and manufacturers reports incomplete technical information. In the continuous optimization mechanism, the observable variable refers to the interaction term for complete technical input information of the R&D institution and complete technical output information of the manufacturers. The linear term and quadratic term for the observable variable are selected as dependent variables for regression analysis. By analyzing the relationship between the linear term and quadratic term for independent variables and those for dependent variables, the equilibrium solution of the independent variables is solved, so as to obtain the technical information required for the optimal IFAE. By comparing the differential effects of incomplete technical information and complete technical information between the single optimization mechanism and the continuous optimization mechanism during the allocation of innovation factors, the research found that how the R&D institution and the manufacturers chose information strategies when their goals were consistent.

**Table 8:** Optimization mechanisms of IFAE and equilibrium analysis.

Industry	single optimization mechanisms									Continuous optimization mechanism		
	Ⅰ			Ⅱ			Ⅲ			IIIO		
	Quadratic term	Linear term	Equilibrium point	Quadratic term	Linear term	Equilibrium point	Quadratic term	Linear term	Equilibrium point	Quadratic term	Linear term	Equilibrium point
	(1)	(2)	(3)	(4)	(5)	(6)	(7)	(8)	(9)	(10)	(11)	(12)
Software development industry	−0.1182	3.1705	13.4116	−0.1294	2.9281	11.3141	−0.1472	2.2938	7.7914	−0.0995	1.5216	7.6462
	(0.0728)	(0.4192)		(0.5091)	(0.9477)		(0.4171)	(0.9022)		(0.5069)	(1.6111)	
Electrical equipment industry	−0.1838	1.7442	4.7448	−0.2491	2.8544	5.7294	−0.2051	3.0894	7.5314	−0.0637	0.5172	4.0597
	(0.1865)	(1.6111)		(0.1163)	(1.0474)		(0.0901)	(1.0039)		(0.1529)	(1.2027)	
Electronic components industry	−0.0393	2.6222	33.3613	−0.17	2.8702	8.4418	−0.1922	2.775	7.219	−0.2229	3.0865	6.9235
	(0.0153)	(0.8639)		(0.1613)	(1.6813)		(0.1280)	(1.6542)		(0.2115)	(1.9252)	
Auto parts industry	−0.4464	4.0404	4.5255	−0.2999	6.0973	10.1656	−0.0068	0.0854	6.2794	−0.3932	6.9817	8.8781
	(0.2315)	(1.7239)		(0.0807)	(1.1808)		(0.0384)	(0.9143)		(0.1058)	(1.3521)	
Communication equipment industry	−0.5935	16.3075 (0.7744)	13.7384	−0.1643	3.4408 (1.4117)	10.4711	−0.0015	0.0129 (0.7285)	4.3000	−0.2154	3.9399 (1.6165)	9.1455
	(0.2108)			(0.0793)			(0.0191)			(0.1040)		
Pharmaceuticals & biotech industry	−0.1487	2.8010	9.4183	−0.1487	2.3951	8.0535	−0.0050	0.1514	15.1440	−0.1950	2.7426	7.0323
	(0.1220)	(2.0320)		(0.1309)	(1.2700)		(0.0127)	(0.4383)		(0.1716)	(1.4543)	

According to the results of empirical analysis, in the single optimization mechanism and the continuous optimization mechanism for the six industries, the coefficients of the quadratic terms are negative, indicating that the IFAE has the highest value. The realization of the R&D institution's goal of benefit maximization and the acquisition of sufficient technical input information by the manufacturers necessitate the optimization of the allocation of innovation factors, which will lead to a great potential for the application of technical information. As Column 3 in Table 8 shows, the equilibrium points of technical information to maximize the IFAE are respectively 13.4116, 4.7448, 33.3613, 4.5253, 13.7384 and 9.4183 in the case of incomplete technical input information and incomplete technical output information for the six industries. Columns 6 and 9 in Table 8 show that under the condition of the interaction term for incomplete technical input information and complete technical output information and the one for complete technical input information and incomplete technical output information for the six industries, the equilibrium points of technical information for the optimal IFAE are respectively 11.3141, 5.7294, 8.4418, 10.1656, 10.4711, 8.0535, 7.7914, 7.5314, 7.2190, 6.2794, 4.4483 and 15.1400. As a result, in the single optimization mechanism of IFAE, the software development industry, as the R&D institution, and the electronic components industry and the communication equipment industry, as the manufacturers, will choose different technical information-related strategies. The equilibrium points of the interaction between incomplete technical input information and incomplete technical output information are higher than those of the other two interaction terms. Incomplete technical information plays a greater role in improving the IFAE. Therefore, the above three industries may also recognize and identify more valuable technical information from a relatively large amount of incomplete technical input information and incomplete technical output information. When the R&D institution or manufacturers provides complete technical information, it will further enhance the information quality and optimize the information structure, so that a relatively small amount of information can gradually optimize the IFAE and both of them give priority to providing complete technical information [27]. According to the research findings, the electrical equipment industry, auto parts industry and pharmaceuticals & biotech industry are characterized by stronger intensity in technology, rapid diffusion of technical achievements in the form of knowledge and information, and a low proportion of incomplete technical input information and incomplete technical output information, and complete technical information changed from technical input information and technical output information will play a more significant role in the optimization of IFAE.

Column 12 in Table 8 indicates that in the continuous optimization mechanism, the incomplete technical input information of the R&D institution and the incomplete technical output information of the manufacturers are gradually transformed into complete technical information, and the IFAE is thus significantly improved. For the six industries, the equilibrium points of the interaction terms for complete technical input information and complete technical output information in the case of optimal IFAE are respectively 7.6462, 4.0597, 6.9235, 8.8781, 9.1455 and 7.0323. The regression results show that the equilibrium points of the interaction terms for the software development industry, electrical equipment industry, electronic components industry and pharmaceuticals & biotech industry are lower than those in the three single optimization mechanisms. This means the full use of complete technical information, which is conducive to improving the structure of the allocation of innovation factors and optimizing the input-output ratio of innovation factors. However, the equilibrium points of interaction terms for the auto parts industry and communication equipment industry are located between those in the three single optimization mechanisms. Improving the IFAE depends on the R&D institution's reporting of complete technical input information, the manufacturers' continuous reporting of complete technical output information, and smaller impact of other factors in the process of technical information absorption and transformation, thus contributing to smoother technical information channels and significantly higher IFAE. The results of empirical analysis verify hypothesis 3.

## 6 Conclusions and prospects

According to the conclusion, when the R&D institution chooses complete technical input information or incomplete technical input information, and manufacturers choose complete technical output information or incomplete technical output information, the IFAE will be significantly different. With regard to different ways of realization, the results produced by the single optimization mechanism and the continuous optimization mechanism of IFAE will be varied. In the allocation of innovation factors, the R&D institution and the manufacturers share the same goal of benefit maximization, and give priority to the reporting of complete technical input information and complete technical output information. In terms of the single optimization mechanism, encouraged by the shared goal, the R&D institution and the manufacturers gradually transform incomplete technical information into relatively more complete technical information and complete technical information, and the IFAE gradually reaches the optimal state, which is realized by the continuous optimization mechanism on the basis of a large number of single optimization mechanisms.

This paper mainly reveals that in the dynamic transformation of incomplete technical information into relatively more complete technical information and finally complete technical information, R&D institutions and manufacturers choose different information strategies to make the single optimization mechanism and continuous optimization mechanism of IFAE tend to be optimal. However, the changes in technical information during the input and output processes of innovation factors are characterized by significant randomness, and may be determined and analyzed only on the basis of the formation probability of complete technical information. The detailed analysis of technical information, the realization of transmission paths and the measurement of the uncertainty of technical innovation efficiency are possible topics for deeper research in the future. Based on the above conclusions, the following recommendations are proposed:

Firstly, governments need to improve the laws and regulations on science and technology to strengthen the protection for intellectual property rights and reflect the value of technical information. In terms of generation, identification, transformation and application of all kinds of technical information, more efforts should be made to fully protect the legitimate rights and interests of owners, encourage all kinds of innovators to invest more resources to enhance original innovation and integrated innovation, acquire greater technical information increment, and obtain more sufficient technical information in the R&D direction, the selection of routes, the value of elements, the allocation of innovation factors, etc.

Secondly, greater support for technology research and development, and collaborative innovation based on the combined efforts of enterprises, universities and research institutions should be provided. The latter streamlines the processes of technical information transmission, reduces the transaction costs of technical achievements, and promotes the flow and transformation of technical information. It is necessary to give full play to technologies in the allocation of innovation resources, reduce administrative dominance and departmental barriers, highlight the characteristics and laws of technical information carriers in the process of the transformation of technical achievements, and promote the industrialization of technical achievements.

Thirdly, it is essential to promote the continuous upgrading of high-tech industries and give full play to enterprises as the key players in technical innovation. The dominant position of enterprises should be presented by the title to ownership, disposal and profitability of technical information. More investments should be made to give full play to the technical information distribution function of corporate R&D centers, support and guide SMEs to acquire more adequate technical information, and accelerate the construction of a number of R&D centers, thus helping technical information truly become a pivot for the production, incubation and application of achievements, and contributing to the rapid promotion and application of technical R&D practical achievements.

The innovation of this study is that it compares and analyzes the different paths of single optimization and continuous optimization of the IFAE. However, under the premise of relatively more complete technical information and complete technical information, further exploratory research is needed to achieve the optimal efficiency of innovation factor allocation in practice, especially focusing on in-depth research on implementable specific policy designs.

## Funding statement

Mr. Linsheng Yang was supported by the Soft Science Research Project in Zhejiang Province[2022C35118].

## Data availability statement

Data will be made available on request.

## CRediT authorship contribution statement

**Linsheng Yang:** Writing – review & editing, Writing – original draft, Visualization, Validation, Supervision, Resources, Project administration, Methodology, Investigation, Funding acquisition, Data curation, Conceptualization. **Bing Jiang:** Writing – review & editing, Writing – original draft, Validation, Software, Methodology, Investigation, Data curation. **Qi Xu:** Writing – review & editing, Writing – original draft, Validation, Software, Resources, Methodology, Investigation, Data curation.

## Declaration of competing interest

We declare that we have no financial and personal relationships with other people or organizations that can inappropriately influence our work, there is no professional or other personal interest of any nature or kind in any product, service and/or company that could be construed as influencing the position presented in, or the review of, the manuscript entitled.
